# Population-based cohort study examining the association between weight loss and pulmonary tuberculosis in adults

**DOI:** 10.1051/bmdcn/2018080212

**Published:** 2018-05-28

**Authors:** Shih-Wei Lai, Cheng-Li Lin, Kuan-Fu Liao

**Affiliations:** 1 College of Medicine, China Medical University Taichung 404 Taiwan; 2 Department of Family Medicine, China Medical University Taichung 404 Taiwan; 3 Management Office for Health Data, China Medical University Hospital Taichung 404 Taiwan; 4 College of Medicine, Tzu Chi University Hualien 970 Taiwan; 5 Department of Internal Medicine, Taichung Tzu Chi General Hospital Taichung 427 Taiwan

**Keywords:** Cohort study, Weight loss, Pulmonary tuberculosis, Taiwan National Health, Insurance Program

## Abstract

Background/Purpose: Little research is currently available on the relationship between weight loss and pulmonary tuberculosis in Taiwan. This study aimed to evaluate whether weight loss is an early clinical feature of pulmonary tuberculosis in Taiwan.

Method: This population-based retrospective cohort study was conducted using the Taiwan National Health Insurance Program database. There were 6051 subjects aged 20 to 84 years with newly diagnosed weight loss from 2000 to 2012 as the weight loss group, and 24081 randomly selected subjects without weight loss from the same period as the non-weight loss group. The weight loss and the non-weight loss groups were matched by sex, age, and comorbidities. The incidence of pulmonary tuberculosis at the end of 2013 was evaluated in both groups. A multivariable Cox proportional hazards regression model was used to evaluate the hazard ratio (HR) and 95% confidence interval (CI) for association of pulmonary tuberculosis with weight loss.

Results: The incidence of pulmonary tuberculosis was 15.2-fold higher in the weight loss group than in the non-weight loss group during the first 3 months of follow-up (22.8 *vs*. 1.50 per 1000 person-years, 95% CI 13.7, 16.9). After adjusting for covariables, the subsequent HR of pulmonary tuberculosis was 2.36 for the weight loss group (95 % CI 1.88, 2.97), compared with the non-weight loss group.

Conclusion: Although our finding is not novel, it does support the notion that weight loss is significantly associated with increased hazard of pulmonary tuberculosis in Taiwan. The risk was found to be particularly high during the first 3 months of follow-up.

## 1. Introduction

Tuberculosis is still a global public health problem due to its high prevalence. World Health Organization reported that there were 9.6 million persons stricken with tuberculosis in 2014. [1] From a view of preventive medicine, early detection and early intervention are important for the eradication of tuberculosis. Previous case-series studies have shown that cough, chest pain, hemoptysis, fever, night sweats, and weight loss were common symptoms in patients with pulmonary tuberculosis. [2-4]

Weight loss is clinically defined as the loss of 5% or more of one’s original body weight within a 6-month period, and it should be further investigated. [5, 6] Weight loss is a common clinical finding frequently associated with severe underlying conditions, including psychiatric disorders, cancer, gastrointestinal disorders, and others. [7-9]

Although this topic has been well investigated in previous work, [2-4] to the best of our knowledge, no systematic research has investigated the association of pulmonary tuberculosis with weight loss in Taiwan. If weight loss is an early clinical feature of pulmonary tuberculosis, physicians can detect pulmonary tuberculosis earlier in these high-risk patients. And thus, early intervention can be performed. Therefore, in this study we aimed to evaluate (1) whether weight loss is associated with pulmonary tuberculosis, and (2) how soon pulmonary tuberculosis is diagnosed after presentation of weight loss.

## 2. Methods

### 2.1. Study design and data source

The study design and data source were adapted from previous studies. [10-12] Briefly, Taiwan is an independent country with more than 23 million residents. [13-16] This population-based retrospective cohort study was conducted using the Longitudinal National Health Insurance Research Database, which contains 1,000,000 beneficiaries randomly sampled from the Taiwan National Health Insurance Program. The program was launched in March 1, 1995, and it covers about 99.6% of 23 million residents living in Taiwan. [17]

### 2.2. Study subjects

We selected 6051 subjects aged 20 to 84 years with newly diagnosed weight loss from 2000 to 2012 as the weight loss group (the International Classification of Diseases (ICD) 9th Revision, ICD-9 codes 783.21). To increase statistical power, for each subject with weight loss, 4 subjects without weight loss were randomly selected as the non-weight loss group. The index date was defined as the date of subjects being diagnosed with weight loss. The index date for non-weight loss subjects was randomly appointed to a month and a day with the same index year as the weight loss subjects matched. Subjects with weight loss and with non-weight loss were matched based on sex, age (every 5-year interval), comorbidities, and the same index year. Subjects with a history of pulmonary tuberculosis before the index date or subjects being diagnosed with pulmonary tuberculosis on the index date were excluded from the study. The non-weight loss group should have been 24204 subjects. Due to strict exclusion and inclusion criteria, only 24081 subjects could be matched in the nonweight loss group.

### 2.3. Comorbidities

Comorbidities which could be potentially related to pulmonary tuberculosis were included as follows: alcohol-related diseases, chronic kidney disease, chronic obstructive pulmonary disease, diabetes mellitus, human immunodeficiency virus infection, gastrectomy, pneumoconiosis, as well as chronic liver diseases including cirrhosis, hepatitis B infection, hepatitis C infection, and other chronic hepatitis. All comorbidities were diagnosed based on ICD-9 codes. The validity of ICD-9 codes has been well tested in previous studies. [18-22]

### 2.4. Main outcome

The main outcome was a new diagnosis of pulmonary tuberculosis (ICD-9 codes 010, 011, 012, and 018) during the follow-up period. All study subjects were followed until they were diagnosed with pulmonary tuberculosis or 2013 ended, whichever came first.

### 2.5. Statistical analysis

The distributions of sex, age, and comorbidities were compared between the weight loss and non-weight loss groups using a *Chi*-square test for categorized variables, and a *t*-test for continuous variables. The incidence of pulmonary tuberculosis was estimated as the event number of pulmonary tuberculosis identified during the follow-up time, divided by the total follow-up person-years for each group. In the beginning, all variables were examined in a univariable model. Next, variables which were found to be statistically significant in a univariable model were further examined in a multivariable model. A multivariable Cox proportional hazards regression model was used to estimate the hazard ratio (HR) and 95 % confidence interval (CI) for the association of pulmonary tuberculosis with weight loss. All analyses were performed using the SAS software version 9.2 (SAS Institute Inc., Carey, North Carolina, USA). The results were considered statistically significant when two-tailed *P* values were less than 0.05.

## 3. Results

### 3.1. Baseline characteristics of the study population

Table 1 discloses the baseline information of the study population. There were 6051 subjects in the weight loss group and 24081 subjects in the non-weight loss group, with similar distributions of sex and age. The mean ages (standard deviation) of the study subjects were 54.3 (16.4) years for the weight loss group and 54.0 (16.5) years for the non-weight loss group (*t*-test, *P* = 0.23). The weight loss group had a higher proportion of gastrectomy than the non-weight loss group (*Chi*-square test, 0.46% *vs*. 0.29%, *P* = 0.04). There was no statistical significance of the other comorbidities between the weight loss and non-weight loss groups (*Chi*-square test, *P* > 0.05).

**Table 1:** Baseline characteristics between the weight loss group and the non-weight loss group.

	Weight loss N = 6051		Non-weight loss N = 24081		
Characteristic	n	(%)	n	(%)	*P* value*
Sex					0.98
Female	2904	(48.0)	11562	(48.0)	
Male	3147	(52.0)	12519	(52.0)	
Age group (years)					0.95
20-39	1339	(22.1)	5371	(22.3)	
40-64	2934	(48.5)	11663	(48.4)	
65-84	1778	(29.4)	7047	(29.3)	
Age (years), mean ± (standard deviation)†	54.3 ± 16.4	54.0 ± 16.5	0.23
Baseline comorbidities					
Alcohol-related diseases	446	(7.37)	1742	(7.23)	0.71
Chronic kidney disease	202	(3.34)	770	(3.20)	0.58
Chronic liver diseases	1672	(27.6)	6613	(27.5)	0.79
Chronic obstructive pulmonary disease	1178	(19.5)	4654	(19.3)	0.80
Diabetes mellitus	522	(8.63)	2041	(8.48)	0.71
Human immunodeficiency virus infection	4	(0.07)	14	(0.06)	0.22
Gastrectomy	28	(0.46)	70	(0.29)	0.04
Pneumoconiosis	54	(0.89)	160	(0.66)	0.06

Data are presented as the number of subjects in each group with percentages given in parentheses.

* *Chi*-square test,

† *t*-test comparing subjects with and without loss.

### 3.2. Incidence of pulmonary tuberculosis of the study population stratified by sex, age, and follow-up period

Table 2 discloses that the overall incidence of pulmonary tuberculosis was 2.32-fold greater in the weight loss group than in the non-weight loss group (3.50 *vs*. 1.51 per 1000 person-years, 95% CI 2.14, 2.52). The incidences of pulmonary tuberculosis, as stratified by sex, age, and follow-up period, were all higher in the weight loss group than those in the non-weight loss group. The section of the weight loss group aged 65 to 84 years had a higher incidence of pulmonary tuberculosis (7.83 per 1000 person-years). Also, the weight loss group had the highest incidence of pulmonary tuberculosis (22.8 per 1000 person-years) in the first 3 months of follow-up.

**Table 2:** Incidence of pulmonary tuberculosis estimated by sex, age, and follow-up period between weight loss group and non-weight loss group.

	Weight loss				Non-weight loss					
Variable	N	Event	Person-years	Incidence†	N	Event	Person-years	Incidence†	IRR*	(95% CI)
All	6051	115	32827	3.50	24081	209	138630	1.51	2.32	(2.14, 2.52)
Sex										
Female	2904	36	16360	2.20	11562	61	67358	0.91	2.43	(2.16, 2.73)
Male	3147	79	16467	4.80	12519	148	71271	2.08	2.31	(2.07, 2.58)
Age group (years)										
20-39	1339	9	8003	1.12	5371	15	32608	0.46	2.44	(2.03, 2.94)
40-64	2934	41	16521	2.48	11663	67	68377	0.98	2.53	(2.25, 2.85)
65-84	1778	65	8303	7.83	7047	127	37644	3.37	2.32	(2.02, 2.67)
Follow-up period (months)										
< 3	6051	34	1495	22.8	24081	9	6009	1.50	15.2	(13.7, 16.9)
≥ 3	5902	81	31333	2.59	23995	200	132621	1.51	1.71	(1.58, 1.87)

† Incidence: per 1000 person-years.

* IRR (incidence rate ratio): weight loss *vs*. non-weight loss. (95% confidence interval)

### 3.3. Association of pulmonary tuberculosis with weight loss

Table 3 discloses the association of pulmonary tuberculosis with weight loss. Variables found to be statistically significant in a univariable model were further examined in a multivariable model. After adjusting for covariables, the multivariable Cox proportional hazards regression model disclosed that the adjusted HR of pulmonary tuberculosis was 2.36 for the weight loss group (95 % CI 1.88, 2.97), compared with the non-weight loss group.

**Table 3:** Cox model measured hazard ratio and 95% confidence interval of pulmonary tuberculosis associated with weight loss and comorbidities.

	Crude		Adjusted*	
Variable	HR	(95% CI)	HR	(95% CI)
Sex (male *vs*. female)	2.22	(1.75, 2.82)	1.98	(1.56, 2.53)
Age (per one year)	1.05	(1.04, 1.06)	1.04	(1.04, 1.05)
Weight loss	2.31	(1.84, 2.90)	2.36	(1.88, 2.97)
Baseline comorbidities (yes *vs*. no)				
Alcohol-related diseases	1.49	(1.03, 2.15)	1.34	(0.92, 1.96)
Chronic kidney disease	3.15	(2.12, 4.67)	1.67	(1.12, 2.50)
Chronic liver diseases	0.99	(0.78, 1.27)	-	-
Chronic obstructive pulmonary disease	2.80	(2.24, 3.50)	1.45	(1.13, 1.85)
Diabetes mellitus	1.94	(1.42, 2.64)	1.19	(0.87, 1.63)
Human immunodeficiency virus infection	11.4	(2.85, 45.8)	28.0	(6.88, 114.2)
Gastrectomy	2.13	(0.53, 8.55)	-	-
Pneumoconiosis	2.09	(0.78, 5.59)	-	-

* Variables which were found to be statistically significant in a univariable model were further examined in a multivariable model. Adjusted for sex, age, alcohol-related diseases, chronic kidney disease, chronic obstructive pulmonary disease, diabetes mellitus, and human immunodeficiency virus infection.

### 3.4. Association of pulmonary tuberculosis stratified by weight loss and comorbidities

Table 4 discloses the association of pulmonary tuberculosis stratified by weight loss and comorbidities. To reduce the potential confounding effects of the comorbidities studied, as a reference of subjects without weight loss and without comorbidities, the adjusted HR of pulmonary tuberculosis was 3.60 for subjects with weight loss alone and without comorbidities (95% CI 2.57, 5.05). This finding indicates that even without the presence of comorbidities, weight loss alone is significantly associated with the increased hazard of pulmonary tuberculosis.

**Table 4:** Risk of pulmonary tuberculosis stratified by weight loss and comorbidities.

Variable		Event	Person-years	Incidence†	Adjusted HR* (95% CI)
Weight loss	Any comorbidity&				
No	No	74	97324	0.76	(Reference)
No	Yes	135	41305	3.27	2.47 (1.84, 3.32)
Yes	No	62	23232	2.67	3.60 (2.57, 5.05)
Yes	Yes	53	9595	5.52	4.19 (2.91, 6.02)

^†^ Incidence rate: per 1000 person-years.

* Adjusted for sex and age.

^&^ Comorbidities including alcohol-related disease, chronic kidney disease, chronic obstructive pulmonary disease, diabetes mellitus, and human immunodeficiency virus infection.

## 4. Discussion

In this population-based retrospective cohort study, we found that the overall incidence of pulmonary tuberculosis was 2.32- fold higher in the weight loss group than in the non-weight loss group. Its incidence was particularly high during the first 3 months of follow-up (incidence rate ratio of 15.2; 22.8 *vs*. 1.50 per 1000 person-years, Table 2). Because subjects with a history of pulmonary tuberculosis before the index date or subjects being diagnosed with pulmonary tuberculosis on the index date were excluded from the study, we think that weight loss substantially occurred before the new diagnosis of pulmonary tuberculosis.

The causal relationship between weight loss and pulmonary tuberculosis is theoretically acceptable. From a view of the high quality of Taiwan’s medical system, it is reasonable to take less than 3 months to make a confirmed diagnosis of pulmonary tuberculosis after the onset of tuberculosis-related symptoms.

In a multivariable-adjusted analysis, compared with no weight loss, weight loss was associated with an increased hazard of pulmonary tuberculosis (adjusted HR 2.36, 95% CI 1.88, 2.97). In a sub-analysis, it was not necessary for this hazard to be accompanied by comorbid conditions. Weight loss alone remains significantly associated with an increased hazard of pulmonary tuberculosis. Based on the above discussion, we think that weight loss might be an early clinical feature of pulmonary tuberculosis. We would like to highlight that a high index of suspicion is needed to detect pulmonary tuberculosis early. Physicians should consider the possibility of pulmonary tuberculosis when people present with weight loss and other tuberculosis-related symptoms. Thus, early treatment of pulmonary tuberculosis can be undertaken with these high-risk patients.

The study has several limitations. First, due to the inherent limitation of the database used, other tuberculosis-related symptoms, such as cough, chest pain, hemoptysis, fever, and night sweats, were not recorded. [2-4] We could not differentiate whether patients with weight loss also had other relevant symptoms toms. On the other hand, some patients not only had cough and chest pain, *et al*., but also had weight loss. These patients only talked about cough and chest pain *et al*., but did not mention their weight loss. Therefore, weight loss was not coded with ICD-9 code 783.21 by these patients’ attending physicians. Only 6051 patients with weight loss were identified during the long study period of this population-based study. The number is too limited and not representative of patients with weight loss. Many patients categorized in the not-weight loss group may have actually had weight loss. This underestimates the prevalence of weight loss. Second, cigarette smoking should be added as a confounder. Due to the same limitation, the status of smoking was not recorded. Only the ICD-9 code of chronic obstructive pulmonary disease could be used for analysis. Third, due to the same limitation, there was no record on weight loss being intentional or unintentional. Meanwhile, it must be noted that people seeking medical consultation for weight loss are those worried about the causes of their weight loss. Therefore, we are confident that their weight loss was unintentional. Fourth, due to the same limitation, the magnitude and duration of weight loss in the study cannot be clearly defined. Only the ICD-9 code of weight loss could be used for analysis. Similarly, there was no record on the culture and acid-fast stain for tuberculosis. Only the ICD-9 code of pulmonary tuberculosis could be used for analysis. Fifth, because no other study can be compared with our study, the accuracy of using the ICD-9 code for the diagnosis of weight loss cannot be validated. Yet, due to the incidence of pulmonary tuberculosis being higher in the weight loss group than in the non-weight loss group (incidence rate ratio 2.32, Table 2), we are confident about the accuracy of using the ICD-9 code for the diagnosis of weight loss in the study. It is thus not likely prone to bias. That is, as long as the ICD-9 code 783.21 is coded, people always have weight loss.

Although this work is largely confirmatory and a sufficient number of studies have already been published on the topic, to the best of our knowledge, this is the first population-based cohort study investigating the association of pulmonary tuberculosis with weight loss in Taiwan. It adds to the knowledge of evidence-based medicine in Taiwan. We used a well-organized national database to test the association of pulmonary tuberculosis with weight loss. Several potential comorbidities have been controlled to reduce the bias. The manuscript is well written. The introduction is completely supported by the results, and the findings are pertinent.

Although the finding is not novel, in this study weight loss has been found to be significantly associated with an increased hazard of pulmonary tuberculosis in Taiwan. The risk is particularly high during the first 3 months of follow-up. We think weight loss might be an early clinical feature of pulmonary tuberculosis. Physicians should consider the possibility of pulmonary tuberculosis when people present with weight loss and other tuberculosis-related symptoms.
